# Current concepts in the pathogenesis of traumatic temporomandibular joint ankylosis

**DOI:** 10.1186/1746-160X-10-35

**Published:** 2014-09-04

**Authors:** Ying-Bin Yan, Su-Xia Liang, Jun Shen, Jian-Cheng Zhang, Yi Zhang

**Affiliations:** 1 Department of Oral and Maxillofacial Surgery, Tianjin Stomatological Hospital, 75 Dagu Road, Heping District, Tianjin 300041, PR China; 2 Department of Operative Dentistry and Endodontics, Tianjin Stomatological Hospital, 75 Dagu Road, Heping District, Tianjin 300041, PR China; 3 Department of Oral and Maxillofacial Surgery, Peking University School and Hospital of Stomatology, 22 Zhongguancun Nandajie, Haidian District, Beijing 100081, PR China

**Keywords:** Trauma, Temporomandibular joint, Ankylosis, Pathogenesis, Review

## Abstract

Traumatic temporomandibular joint (TMJ) ankylosis can be classified into fibrous, fibro-osseous and bony ankylosis. It is still a huge challenge for oral and maxillofacial surgeons due to the technical difficulty and high incidence of recurrence. The poor outcome of disease may be partially attributed to the limited understanding of its pathogenesis. The purpose of this article was to comprehensively review the literature and summarise results from both human and animal studies related to the genesis of TMJ ankylosis.

## Introduction

Temporomandibular joint (TMJ) ankylosis is often described as either fibrous or bony, and, in traditional opinion, fibrous ankylosis can progress into bony ankylosis [1]. The most common aetiology of TMJ ankylosis is trauma, mainly condylar fracture [2,3]. Although a close relationship exists between condylar fracture and TMJ ankylosis [4], the pathogenesis of the disease remains ill-defined [5], and very few publications have investigated the issue.

In this review, focusing on bony ankylosis, we will describe the current understanding of the clinical, imaging and pathological features of the disease. Then, we will discuss the underlying condition of the disease based on evidence from both animal and human studies. The hypotheses regarding its pathogenesis will be exhaustively summarised and critically evaluated. We will also introduce the advances of cellular and molecular mechanisms of new bone formation in bony ankylosis, and provide new perspectives to prevent the disease.

### Clinical and imaging features

The onset of disease usually occurs in children under 10 years [6] with a roughly equal gender involvement [7]. A progressive reduction in jaw movement is the main clinical presentation. It should be noted that most patients can still move their jaws slightly at the initial examination, and complete limitation of mouth opening is rare [8,9], which means that opening movement exists throughout the entire course of bony ankylosis. Generally, the formation of bony ankylosis takes a long time, ranging from several months to several decades after the occurrence of injury [10-12].

**Figure 1:**
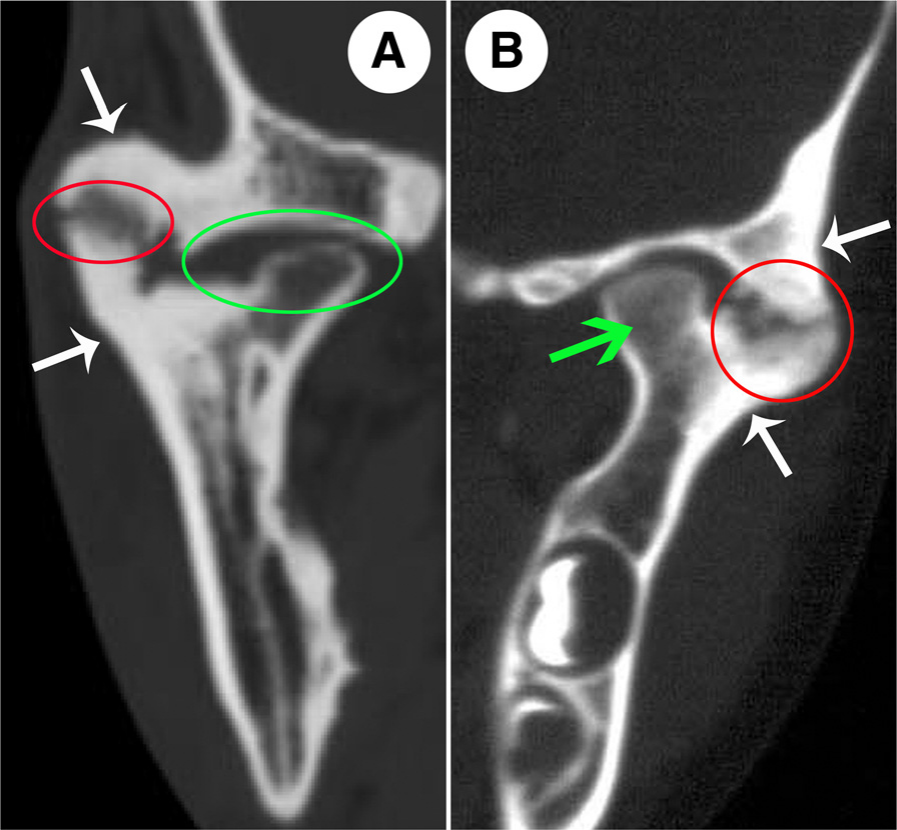
**The computerized tomographic features of TMJ bony ankylosis.** In **A**, the red circle refers to the bony fusion area located in the lateral part of the joint, in this area, a radiolucent zone can be observed. The green circle refers to the atrophic condylar head and rudimentary joint space located in the medial part of the joint. In **B**, the red circle shows osteosclerosis in the bony fusion area, and a radiolucent zone can also be observed in this area. The green arrow indicates that bone mineral density and morphology of the bone marrow cavity in the non-bony fusion area were normal. In **A** and **B**, the white arrows indicate excessive bone formation around the joint. Note in the radiolucent zone, no scattered calcified dots can be found.

According to the literature and our careful observations, the computerized tomographic features of bony ankylosis can be summarised as follows: ① bony fusion is mostly located in the lateral part of the joint, whereas the atrophic condylar head and rudimentary joint space can often be seen in the medial part of the joint [13-15] (Figure 1A). ② In the bony fusion area, the glenoid fossa and condyle demonstrate osteosclerosis with a decreased or absent bone marrow cavity. In the non-bony fusion area of the joint, bone mineral density and the morphology of the bone marrow cavity are similar to the normal bone [8,16] (Figure 1B). ③ For the overwhelming majority of patients, the deformed TMJ is characterized not only by the enlarged condyle, thickened temporal bone and excessive bone formation, but also by a radiolucent zone in the bony fusion area [8,14,16,17] (Figure 1A and B). ④ No scattered calcified dots can be found in the radiolucent zone, demonstrating that the ossification is occurring with the existing bones [8,14,16] (Figure 1A and B).

### Pathology

#### Human data

According to the imaging features mentioned above, the radiolucent zone representing the fusion interface of the 2 traumatic articular surfaces should be the focus of histological examinations. However, due to the difficulty in operating, the specimens available for histological analysis from patients are limited to the ankylosed condyle or incomplete tissue from the radiolucent zone; intact ankylosed joints may only be obtained by autopsy.

Previously published data on the histological manifestations of traumatic TMJ ankylosis are very rare. In 1957, through examining a post-mortem specimen of partial fibrous ankylosis secondary to injury, Blackwood [18] found an enlarged condyle, a flattened surface of the glenoid fossa, and dense avascular fibrous tissue filling the cavity of the condyle. Sarma and Dave [13] analysed 60 specimens and found that all of the samples were composed of two parts. The non-adhesive part demonstrated an atrophic condylar articular surface, and the bony-adhesive part presented with new bone formation. According to the findings of Wu et al. [10], fibrous ankylosis is shown as fibrous tissue intruding into the bone marrow of the condyle with degeneration of the condylar cartilage, whereas bony ankylosis manifests as new bone formation on the rough ankylotic surface of the condyle with slight bone degeneration.

Recently, Li et al. [19] analysed 10 specimens including 1 of fibrous ankylosis and 9 of bony ankylosis. In particular, to acquire histological information in the radiolucent zone, they carefully protected this part of the tissue during the operation [19]. They found fibrous and cartilaginous tissue in the joint space of fibrous ankylosis. The tissue in the radiolucent zone of bony ankylosis was cartilage and new bone matrix, and bony fusion was formed by new osteophytes progressing towards the centre of the ankylotic mass [19]. They concluded that bony ankylosis was formed by endochondral ossification and osteophyte proliferation [19].

#### Data from animal models

The specimens from patients can only represent one stage of the disease, which is often the end stage. Therefore, the true pathological course is generally vague and nondescript, especially for the early stage. Although an animal model exactly mimicking human disease has not been established to date, the existing models provide useful information for the pathological changes of disease. According to animal models, the typical pathological feature of fibrous ankylosis is abundant fibrous connective tissue occupying the joint space with or without cartilage on the traumatic articular surfaces [20-22]. It is noteworthy that fibro-osseous ankylosis, not fibrous ankylosis, is the intermediate form of bony ankylosis according to animal studies [22]. The histological characteristic of fibro-osseous ankylosis, enabling distinction from fibrous ankylosis, is the presence of plenty of cartilaginous tissue in the joint space [22-24], which ultimately forms the bony bridge between the condyle and the temporal bone, namely bony ankylosis [22].

### The underlying condition of the disease

#### Human data

The reason for the occurrence of traumatic TMJ ankylosis is still a mystery, partly due to the low incidence of ankylosis after TMJ trauma [6,25], and the long latent period between the cause and effect. Laskin [6] generalized the factors related to disease, including the age of the patient, severity of trauma, pattern of condylar fracture, duration of immobilization, and location of the disc. Patients characterized with young [6,26], severe TMJ trauma [6], communited condylar fracture [6,26,27] or sagittal fracture [11,26-28], or those with medially dislocated condylar fracture [14], prolonged immobilization of the mandible [6], and disc displacement [6,28] are prone to developing ankylosis. In addition, close contact of the 2 injured articular surfaces, which results in a shorter distance for bone healing, also plays an important role in the development of ankylosis [11].

#### Data from animal models

Animal studies will contribute to verification of the predisposing factors suggested by clinical observations. The animal models related to traumatic TMJ ankylosis in the past 40 years are summarized in Table 1. Restricted jaw movement is not the determinant factor, but rather the promoting agent for ankylosis [29,30]. Discectomy and injury to both articular surfaces are the prerequisites of TMJ ankylosis [31,32]. For bony ankylosis, Yan et al. [22] emphasized the key role of primary severe trauma to the glenoid fossa through a contrasting experiment because minor damage to the glenoid fossa only led to fibrous ankylosis. Recently, it has been shown in rats that protein-energy malnutrition may be a predisposing factor for TMJ fibrous ankylosis [33].

**Table 1:** Animal models related to traumatic TMJ ankylosis in the past 40 years

**Author**	**Year**	**Animal**	**Age/weight**	**Group**	**n**	**Last Time-point**	**Induction methods**					**Results**
							**Bilateral/ unilateral**	**Condyle**	**Disc**	**Glenoid fossa**	**Others**	
Stevenson [30]	1979	Baboon	Juvenile	1	1	32 weeks	Bilateral	Not handled	Not handled	Not handled	Prolonged immobilization until sacrifice	No ankylosis
			Juvenile	2	1	32 weeks	Bilateral	Bilateral fracture	Not handled	Not handled	Prolonged immobilization until sacrifice	No ankylosis
			Juvenile	3	2	32 weeks	Bilateral	Bilateral fracture	Bilateral discectomy	Not handled	Immobilization for 6 weeks	No ankylosis
			Juvenile	4	2	32 weeks	Bilateral	Bilateral fracture	Bilateral discectomy	Not handled	Prolonged immobilization until sacrifice	Fibrous ankylosis
Markey et al. [34]	1980	Monkey	23–27 months	1	2	1 year	Unilateral	Intracapsular condylar fracture	Not handled	Not handled	Intermaxillary fixation for 10 weeks	No ankylosis
			22–26 months	2	2	8 months	Unilateral	Intracapsular condylar fracture with inversion of condylar head	Not handled	Not handled	Intermaxillary fixation for 10 weeks	No ankylosis
			22–26 months	3	2	6 months	Unilateral	Intracapsular condylar fracture with inversion of condylar head	Discectomy	Removal of temporal surface until bleeding	Intermaxillary fixation for 10 weeks	No ankylosis
Hohl et al. [35]	1981	Monkey	1–2 years	1	2	16 months	Unilateral	Morcellate condylar head	Discectomy	Decorticated	Bone graft in joint space	One was complete ankylosis, the other partial ankylosis.
Ishimaru and Goss [36]	1992	Sheep	Adult/60 kg	1	5	3 months	Unilateral	Removal of fibrous layer and underlying cartilage of the condyle	Not handled	Not handled	No	Osteoarthritis: eburnated condyle with osteophytes, thin or perforated discs, and temporal surface proliferation
Ogi et al. [37]	1996	Sheep	Adult/60 kg	1	4	6 months	Bilateral	Removal of fibrous layer and cartilage of the bilateral condyle	3 months later removal the unilateral disc	Not handled	No	No ankylosis: fibrous repair of the articular surfaces
Yao et al. [38]	1999	Minipig	Young	1	6	6 months	Unilateral	Transverse fracture	Not handled	Not handled	No	TMJ adaptive changes
				2	6	6 months	Unilateral	Longitudinal fracture (namely Sagittal condylar fracture)	Not handled	Not handled	No	No ankylosis: bifid condyle deformity and adhesion between disc and condyle
Miyamoto et al. [32]	1999	Sheep	Adult	1	6	3 months	Unilateral	Exision of 5 mm condylar head	Not handled	Removal of temporal surface until bleeding	No	No ankylosis: fibrous repair of the articular surfaces
				2	6	3 months	Unilateral	Exision of 5 mm condylar head	discectomy	Removal of temporal surface until bleeding	No	Fibrous ankylosis
Miyamoto et al. [39]	2000	Sheep	Adult	1	6	3 months	Unilateral	Exision of 5 mm condylar head	discectomy	Removal of temporal surface until bleeding	No	Fibrous ankylosis
				2	6	3 months	Unilateral	Exision of 5 mm condylar head	discectomy	Removal of temporal surface until bleeding	Insertion of intra-articular bone fragment	More extensive fibrous ankylosis with isolated bony island in the joint space
Miyamoto et al. [29]	2000	Sheep	Adult	1	9	3 months	Unilateral	Exision of 5 mm condylar head	discectomy	Removal of temporal surface until bleeding	No	Fibrous ankylosis
				2	9	3 months	Unilateral	Exision of 5 mm condylar head	discectomy	Removal of temporal surface until bleeding	Limit jaw movements by a wire	Fibrous ankylosis was hastened at early stage, but not at advanced stage
Ozten et al. [40]	2004	Guinea pigs	Young/250 g	1	10	2 months	Bilateral /unilateral	Not handled	Not handled	Not handled	Autologous blood injection into joint space	No ankylosis
				2	10	2 months	Bilateral /unilateral	Damage the articular surface by blunt trauma	Preservation of disc	Damage the articular surface by blunt trauma	No	They claimed fibrous tissue formed in the joint, and ankylosis was achieved.
				3	10	2 months	Bilateral /unilateral	Condyle neck fracture	Not handled	Not handled	No	No ankylosis
				4	10	2 months	Bilateral /unilateral	Excision of condyle head	Not handled	Not handled	no	They claimed marked osseous tissue formed, and ankylosis was achieved.
Long and Goss [41,42]	2007	Sheep	2-year-old	1	10	12 weeks	Unilateral	Type B intracapsular condylar fracture	Disc displacement	Not handled	No	Osteoarthritis with progressive changes toward ankylosis
Cheung et al. [23]	2007	Minipig and goat	About 40 kg for minipig, 22 kg for gaots	1	3 + 3	3 months	Bilateral	Exision of 8 mm condylar head	Discectomy on one side, disc preservation on the other side	Not handled on the side of disc preservation, roughed the glenoid fossa on the side of discectomy	Autogenous bone graft on the side of discectomy	Fibrous ankylosis or fibro-bony ankylosis on the side of discectomy, no ankylosis on the side of disc preservation
		Goat	About 27 kg	2	3	3 months	Bilateral	Exision of 8 mm condylar head	Discectomy	Roughed the glenoid fossa	Autogenous bone graft	bony ankylosis on both sides
Porto et al. [20]	2008	Rat	Adult	1	30	90 days	Unilateral	Damaged by a file	Disc removal	Damaged by a file	No	Fibrous ankylosis, no bony bridge was observed
Li et al. [21]	2009	Rat	1-month-old/growing	1	12	12 weeks	Unilateral	Damage to the condylar cartilage with displaced subcondylar head fracture	Damaged the attachments of disc	Not handled	No	Fibrous ankylosis
				2	12	12 weeks	Unilateral	Displaced subcondylar head fracture	Not handled	Not handled	No	No ankylosis
Porto et al. [43]	2011	Rat	Adult	1	18	60 days	Unilateral	Damaged by a file	Disc removal	Damaged by a file	Bone graft in joint space	Fibrous ankylosis, no bony bridge was observed
				2	18	60 days	Unilateral	Damaged by a file	Disc removal	Damaged by a file	Stem cells placed in joint space	Fibrous ankylosis with cartilage, but no bony bridge was observed
Rodrigues et al. [33]	2011	Rat	Adult	1	15	3 months	Unilateral	Not handled	Not handled	Not handled	A hypoprotein diet	Atrophy of the fibrocartilage of the articular surfaces
				2	15	3 months	Unilateral	Condylar fracture	Not handled	Not handled	A hypoprotein diet	Fibrous ankylosis
				3	15	3 months	Unilateral	Condylar fracture	Not handled	Not handled	An ordinary diet	Fracture healing normally
Yan et al. [22]	2013	Sheep	About 20 kg	1	6	6 months	Bilateral	Sagittal condylar fracture	Discectomy on one side, disc preservation on the other side	Not handled on the side of disc preservation, minor damage to the glenoid fossa on the side of discectomy	No	Fibrous ankylosis on the side of discectomy, no ankylosis on the side of disc preservation
			About 20 kg	2	7	6 months	Bilateral	Sagittal condylar fracture	Discectomy on one side, disc preservation on the other side	Not handled on the side of disc preservation, severe damage to the glenoid fossa on the side of discectomy	No	Fibro-bony ankylosis and bony ankylosis on the side of discectomy, no ankylosis on the side of disc preservation

In a sheep model created by Miyamoto et al. [31], discectomy and severe injury to both articular surfaces were performed; however, the outcome was still fibrous ankylosis. The reason why bony ankylosis is not achieved, we believe, may lie in the fact that this group excised a 5 mm condylar head, meaning that the distance for bone healing between the 2 injured articular surfaces was too long. As an illustration for this suggestion, Cheung et al. [23] employed similar induction methods in addition to bone graft in the joint, and achieved bony ankylosis. Bone grafts can promote bony ankylosis because they not only provide osteoconductive scaffold, but also shorten the length of bone healing.

**Figure 2:**
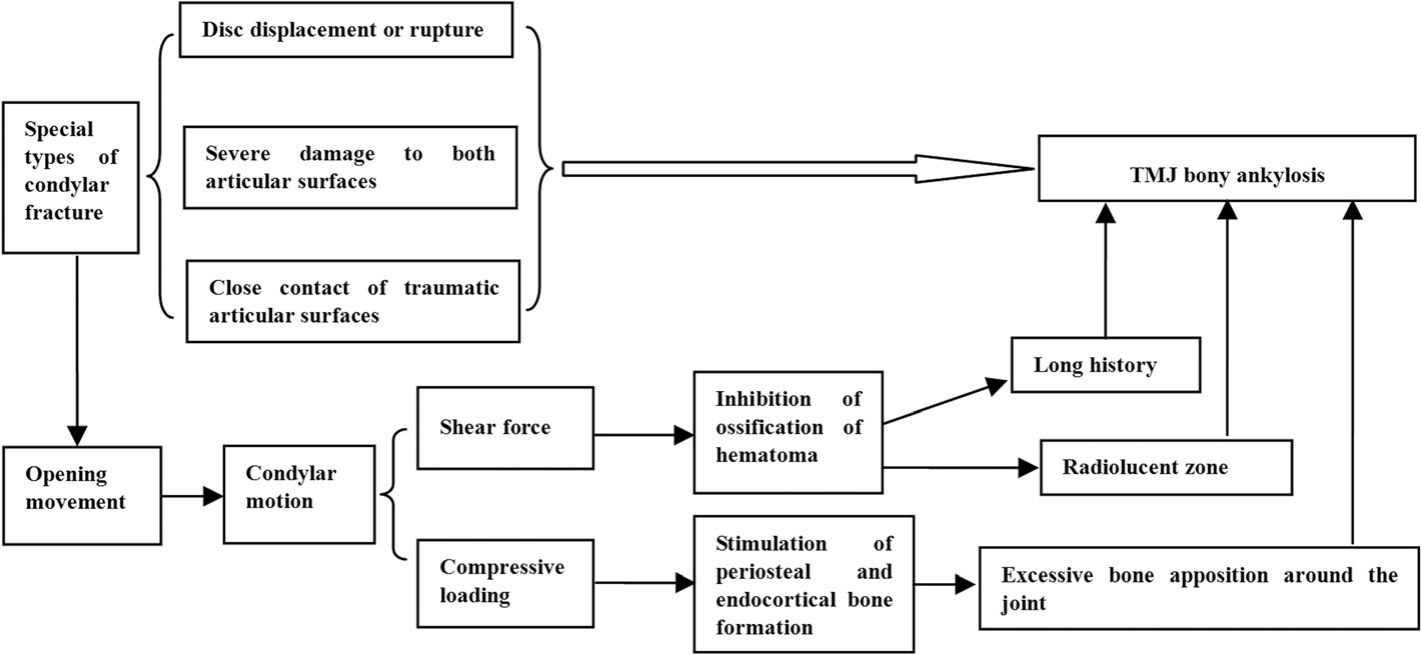
The schematic diagram of our hypotheses.

When the underlying condition is discussed, the conformity between animal models and human disease should be taken into account. There are considerable differences in the TMJ size, anatomy, and function between experimental animals and humans. We must also admit that marked differences exist between disc displacement and discectomy, and between condyle fracture and artificial injury to articular surfaces. In particular, severe experimental injury to the glenoid fossa seems to be obviously deviated from the clinical situations because the fossa is not typically eroded in daily practice. In short, what we have learnt definitely is that bony TMJ ankylosis is incredibly difficult to duplicate in animal models unless resorting to extremes such as grafting in the surgical joint [23] or severe experimental trauma to both of the articular surfaces [22]. However, since the true traumatic microenvironment of TMJ ankylosis in human beings has not been identified, the animal models are invaluable aids to gain insights into the pathogenesis of the disease although none of these exactly mimics the human disease.Taken together, current evidences suggest that the underlying condition of traumatic TMJ bony ankylosis includes disc displacement or rupture, severe damage to both articular surfaces, and close contact of traumatic articular surfaces (See Figure 2).

### The pathogenesis: existing hypotheses and evaluations

#### Intra-articular haematoma

From a classical viewpoint, the pathogenesis of bone formation after trauma is secondary to haemarthrosis [15,44]. Trauma to the condyle can cause disruption of the capsular ligament and adjoining periosteum, resulting in haemarthrosis. When the intra-capsular haematoma following condylar fracture organizes, bone formation can occur from the disrupted periosteum or from metaplasia of non-osteogenic connective tissue [15,44], and bony ankylosis eventually develops. The hypothesis can clearly explain how the bony fusion develops.

It is noteworthy that failure to induce ankylosis by the haemarthrosis experiment [40] cannot negate the rationality of the hypothesis. The injection of blood into the joint space is different from an intra-articular haematoma caused by the impaction of the condylar head against the articular fossa. In the latter, the underlying bone marrow space of the condyle is exposed, which may delivery mesenchymal stem cells (MSCs) into the joint space for osteoblastic differentiation [45,46]. In addition, even simple autologous blood injection into the TMJ can effectively treat chronic recurrent TMJ dislocation through fibrotic changes of the joint [47,48], which indicates that the organization of haematoma secondary to condylar fracture can restrict jaw movement and provide a favourable environment for bony fusion.

However, the hypothesis has flaws. If bony ankylosis is only a simple organization and ossification of an intra-capsular haematoma, then it should be similar to normal fracture healing. However, the history of bony ankylosis is much longer, and, there is still a radiolucent zone in the bony fusion area for most patients [8].

#### Extracapsular haematoma

Ferretti et al. [14] suggest it is not intra-capsular haematoma but extra-capsular haematoma that makes a difference during the development of ankylosis according to the fact that the bony fusion often locates in the juxta-articular area [13]. They state that traumatic TMJ ankylosis is inappropriate tissue differentiation after condylar fracture, and repeated opening movements can cause the disruption of angiogenesis and a failure of osteogenesis. Therefore, sufficient immobility is a prerequisite for ankylosis [14]. In this hypothesis, the inhibitory effect of opening movement on ankylotic bone formation is taken into account. However, most patients with ankylosis did not treat their original TMJ injury by intermaxillary fixation [14]. Additionally, this hypothesis can not yet explain the long history of bony ankylosis.

#### Distraction osteogenesis

Meng et al. [49] consider that distraction osteogenesis of the lateral pterygoid muscle during the healing process of sagittal condylar fracture plays an important role in the genesis of traumatic TMJ ankylosis. However, distraction osteogenesis seems to exist only in the sagittal fracture, not in other fracture types with a high risk of causing ankylosis. Furthermore, new bone formation in distraction osteogenesis always responds to the direction of tensile. Since the tensile from the lateral pterygoid muscle is primarily horizontal, this may partly contribute to the horizontal enlargement of the condyle [50-52]. However, distraction osteogenesis is not associated with vertical bone formation of the condyle and thickening of the temporal bone. Last but not least, the hypothesis can not well explain how the 2 traumatic articular surfaces fuse.

#### Genetic predisposition

Based on the low incidence of ankylosis after condylar fracture [6,25] and the infrequent patients with TMJ ankylosis even after arthroscopy, Hall [53] suggests that it is not trauma but genetic predisposition that is related to traumatic TMJ ankylosis. A recent report described how Shox2-deficiency led to TMJ fibrous ankylosis in mice [54]. In addition, mice with a loss of function mutation in the ank gene (ank/ank mice) not only develop a phenotype of ankylosing spondylitis [55], but also develop fibrous ankylosis in the TMJ [56]. However, whether ANKH, a human homolog of the murine ank gene, is a susceptibility factor for human TMJ ankylosis has not been determined [56]. Recently, studies have revealed that mutations of the PLCB4 and GNAI3 genes cause auriculocondylar syndrome which is characterized by TMJ ankylosis as a common clinical manifestation [57-59].

The core idea of Hall’s hypothesis is that the genesis of traumaic TMJ ankylosis is dependent on the genetic predisposition, rather than the severity of TMJ trauma. However, no family clustering of traumatic TMJ ankylosis has been found to date, which does not support the role of genetic factors in disease susceptibility. This hypothesis can not explain why only unilateral ankylosis occurs for patients with bilateral condylar fractures since the bilateral TMJs of a certain person possess the same hereditary background.

In addition, a better explanation for the low incidence of traumatic TMJ ankylosis may be the lack of underlying condition mentioned above for most patients with TMJ trauma. Our animal model does not support Hall’s hypothesis. A contrasting experiments was performed using the animal model. The results showed that condylar fracture with disc preservation did not induce ankylosis; however, when condylar fracture and discectomy were provided, relatively milder injury to the glenoid fossa could lead to fibrous ankylosis, whereas serious trauma of the glenoid fossa resulted in bony ankylosis [22] (Table 1). Our experiments demonstrated all of the sheep developed bony ankylosis as long as the induced conditions were provided, regardless of the genetic predisposition, indicating that the severity of TMJ trauma determined the outcomes [22].

It is noteworthy that there is marked geographic variation in the perceived frequency of TMJ ankylosis indeed, namely a number of patients with TMJ ankylosis in developing countries and the relative scarcity of this disorder in developed countries [26]. However, the most plausible reason for this phenomenon may be an increased incidence of condylar fractures and unavailability of appropriate care for patients in developing countries [11], rather than different hereditary background.

Taken together, the current evidence suggests that the role of genetic factors in the genesis of traumatic TMJ ankylosis has not yet been identified, and deserved to be further studied.

#### Hypertrophic non-union and its supplement

In a new hypothesis recently proposed by Yan et al. [60], a series of similarities between traumatic TMJ bony ankylosis and hypertrophic non-union, including medical history, aetiology, imaging features, histology, and turnover of disease, were revealed. The hypothesis that the disease course was similar to the hypertrophic non-union was based on the following two prerequisites. Firstly serious TMJ trauma establishes a suitable microenvironment for the bone healing of the 2 articular surfaces, namely creating the underlying condition of bony ankylosis. Secondly, the bone healing of the injured articular surfaces is inhibited by the interference of the opening movement [60]. The hypothesis can easily explain why the incidence of ankylosis secondary to condylar fracture is so low, because very few injured joints can meet the underlying condition of ankylosis. In addition, when taking the opening movement into account, the long clinical course and the radiolucent zone of bony ankylosis can be clearly explained by the hypothesis.

Arakeri et al. [5] considered that the traumatic TMJ ankylosis did not follow the characteristic events of fracture healing because it involved the fusion of 2 different bony surfaces. Indeed, in anatomy, hypertrophic non-union often involves in only one bone, whereas TMJ ankylosis consists of 2 bones and even a disc. However, in biology, we believe that healing between different bony surfaces, such as vascularized bone graft or arthrodesis, is generally the same process as fracture healing. In fact, we have confirmed the similarity between bony ankylosis and fracture healing by histological analysis and molecular examination in a sheep model [22,61,62]. From a broader point of view, arthrodesis, the artificial bony ankylosis, is normal bone healing under the strict fixation of a joint; traumatic TMJ bony ankylosis is the course of hypertrophic non-union under the interference of opening movement; and TMJ fibrous ankylosis, which is postulated to be an independent pathological process different from bony ankylosis [22], can be regarded as atrophic non-union [63].

The hypothesis of hypertrophic non-union can explain the radiolucent zone of bony ankylosis. However, it only points out the phenomenon of the excessive bone apposition around the joint, rather than explaining the causes. Yan et al. [62] therefore proposed a supplementary hypothesis by taking into account the complex mechanical microenvironment after condylar fracture. In this theoretical model, cyclic shear force from the condylar gliding and the dynamic compressive loading from the impact of the condyle against the glenoid fossa are postulated [62]. They suggested that the shear force was the cause of the radiolucent zone, and the increased compressive loading due to disc displacement could stimulate new bone formation around the joint [62]. From this perspective, condylar motion plays dual effects on the bone formation of TMJ bony ankylosis. Their hypotheses can be summarized by the following schematic diagram (Figure 2).

#### Hypercoagulable state of blood

One interesting phenomenon about traumatic TMJ ankylosis is that a few injured joints ankylose; most do not. Recently, Bhatt et al. [64] attributed the low incidence of ankylosis to specific body physiology and the response to trauma. Based on 4 cases who had bilateral traumatic TMJ ankylosis with extrahepatic portal venous obstruction (EHPVO) secondary to protein C deficiency, Bhatt et al. [64] postulated that the hypercoagulability of blood might be a susceptibility factor for TMJ ankylosis.

This hypothesis is very interesting, and potentially provides a reasonable explanation for the low incidence of traumatic TMJ ankylosis. However, not all patients with TMJ ankylosis without EHPVO have hypercoagulable state [64]. In addition, the hypothesis does not consider the underlying condition for traumatic TMJ bony ankylosis. In fact, according to the hypothesis of hypertrophic non-union [60], the reason that most patients with condylar fracture do not develop ankylosis may only be the lack of the underlying condition, as mentioned above.

### Cellular and molecular mechanisms of new bone formation

#### Type of new bone formation

It is well known that 2 different types of new bone formation, endochondral and intramembranous ossification, occur during the embryonic development and postnatal growth. Fracture healing, which is considered to recapitulate the process of skeletal development, takes place mainly through endochondral and partially intramembranous bone formation.

For TMJ bony ankylosis, new bone formation is not ectopic, but orthotopic, because it is in continuity with the existing bones according to the imaging findings mentioned above [8]. Data from animal models [22-24] and human specimens [19,65,66] demonstrate that new bone formation between the 2 articular surfaces is mainly attributable to endochondral ossification, although intramembranous bone formation may also contribute to this [15]. Whether chondroid ossification, a distinctive pattern of bone formation characterized by chondrocyte-like cells in a calcified bone-like matrix [67], occurs in TMJ bony anlykosis is unclear.

#### Cellular and molecular mechanisms

Under physiological circumstances, osteogenesis depends on the osteogenic cells, growth factors and their interactions. For bony ankylosis, the new bone formation is not physiological but pathological, because the continued osteogenesis replaces the normal structure of the articulation and it seems that no remodelling takes place. However, osteoblasts, which are derived from mesenchymal stem cells (MSCs), are the only bone-forming cells. In addition, increasing evidences support the hypothesis that similar signaling molecules and pathways, for example BMP and Wnt signalling, may be employed in both physiological and pathological bone formation [68,69]. Therefore, in the current situation where the pathogenesis of the traumatic TMJ bony ankylosis is unclear, answers for the two following questions will contribute to advancing our understanding about the cellular and molecular mechanisms of the disease. One is where the MSCs participating in ankylotic bone formation are located, and the other is whether BMP/Wnt signalling is involved in ankylotic bone formation.

For the first question, Xiao et al. [70] provided a rational explanation. They consider that, like the hypertrophic non-union tissue [71], the radiolucent zone tissue in bony ankylosis should also contain MSCs. By using ankylosed specimens from 8 patients, they found that the radiolucent zone-related cells possess the properties of MSCs but with lower proliferation and osteogenic differentiation capacity compared to mandibular bone marrow stem cells [70]. Their studies provide cytological evidence for the hypothesis of hypertrophic non-union, and demonstrate that the radiolucent zone may be a potential reservoir of MSCs for ankylosed bone formation.

Wnts are secreted glycoproteins highly conserved between species, and there are at least 19 Wnt ligands [72]. The Wnt pathway plays vital roles in embryonic bone development, postnatal regulation of bone mass and bone regeneration [73-76]. The canonical Wnt signalling is essential for osteoblast lineage differentiation, and mesenchymal precursor lacking canonical Wnt signalling can not differentiate into osteoblast instead of chondrocyte [77].

BMPs are members of the transforming growth factor β (TGF-β) family well known for their osteogenic potential. As the main regulator of chondrocyte proliferation, survival and differentiation, BMP signalling has a remarkable ability to induce endochondral bone formation [78]. BMP2 is necessary for the initiation of frature healing [79,80], BMP4 and BMP7 play an importance role in the late stage of endochondral ossification [81].

The potential roles of BMP/Wnt signalling in traumatic TMJ bony ankylosis were recently studied using animal models or human specimens [61,62,65,66]. Kim et al. [66] found that the hyperplastic chondroid tissues in a human ankylosed sample were positive for BMP-4 but sparse for BMP-2, indicating that BMP signaling may be involved in the ankylosed bone formation through endochondral ossification. However, Pilmane and Skagers [65] demonstrated the lack of BMP2/4 expression in ankylotic bone instead of the rich expression of TGF-β, indicating that bony ankylosis is the disorders of cellular differentiation with compensatory intensification of cellular proliferation.

Based on a reliable animal model, Yan et al. [61,62] demonstrated that BMP and Wnt signalling, which play important roles in bone healing, might be activated during the development of traumatic TMJ bony ankylosis. By exploring the differential expressions of genes regulating bone formation among TMJ fibrous ankylosis, bony ankylosis and condylar fracture healing, they found that the activity of osteogenesis in bony ankylosis was higher than that in fibrous ankylosis, but lower than in condylar fracture [62]. These results provided evidence supporting the hypothesis of hypertrophic non-union at the molecular level. The results indicated that the higher activity of BMP and Wnt signalling in the bony ankylosis compared to fibrous ankylosis was the molecular base leading to continuous new bone formation [62].

### The prevention of bony ankylosis

According to the hypotheses of hypertrophic non-union, once TMJ trauma establishes the microenvironment for the bone healing of the 2 articular surfaces, the development of ankylosis is unavoidable. In this situation, whether the outcome is fibrous or bony ankylosis depends on the severity of the primary TMJ trauma. Relatively milder injury of TMJ leads to fibrous ankylosis, whereas serious TMJ trauma results in bony ankylosis [22]. Therefore, clinically, the fundamental method for the prevention of TMJ ankylosis is to eliminate the underlying condition of ankylosis. For example, when sagittal fracture of condyle with disc displacement occurs, the glenoid fossa may also suffer primary severe trauma, and the microenvironment probably meets the underlying condition of ankylosis. Such patients should be operated upon in a timely manner for reduction and fixation of the condylar fracture and reposition of the displaced disc to avoid the development of ankylosis.One of the important goals of the treatment of TMJ ankylosis is to maintain normal mouth opening. In fact, patients with fibrous ankylosis often open their mouth wider than those with bony ankylosis. According to the hypotheses of hypertrophic non-union, when the underlying condition of ankylosis is to be provided, the mouth opening will exert a dual effect on the new bone formation indeed (Figure 2). However, in the early phase after TMJ trauma, the active jaw-opening exercises can increase the tissue deformation in the joint space and promote the formation of fibrous tissue, thus probably converting bony ankylosis into fibrous ankylosis. Therefore, initiating jaw-opening exercises as soon as possible after condylar fracture is necessary for the prevention of bony ankylosis.

Besides increasing the mechanical instability between the 2 injured articular surfaces through mouth-opening exercises, other methods for inhibiting bone formation or fracture healing, such as non-steroidal anti-inflammatory drugs [82], low-dose irradiation [83,84], antagonists of BMP and Wnt signalling pathways [76,85] and so on, may also be beneficial for the prevention of bony ankylosis. However, like mouth-opening exercises, those means can only convert bony ankylosis into fibrous ankylosis rather than prevent the onset of bony ankylosis.

It is well known that MSCs, possessing the property of pluripotency, play important roles during the course of bone healing. Changing the cell lineage determination of MSCs by manipulating specific transcription factors may be another suitable prophylactic method for bony ankylosis. We believe that FGF21, a key mediator of Peroxisome proliferator-activated receptor-γ (PPARγ) might be such a promising drug [86]. FGF21 can stimulate adipocyte differentiation of the MSCs while suppressing osteoblast differentiation [87,88], thus resulting in the formation of fat pads and the inhibition of new bone formation in the joint space. The fat pads can separate the condyle from the glenoid fossa, serving as physical barrier and mechanical buffer [89,90], ultimately prohibiting the onset of bony ankylosis, and even avoiding the occurrence of fibrous ankylosis.

## Conclusion

The true traumatic microenvironment leading to TMJ ankylosis has not been identified. Although animal models and clinical observations have provided new evidence about the pathogenesis of traumatic TMJ bony ankylosis, the biological events and molecular mechanisms are far from being comprehensively understood. The hypotheses of hypertrophic non-union and its supplement seem to grasp the nature of bony ankylosis and explain the development of the disease. A series of recent clinical and experimental studies have preliminarily verified the hypotheses at the cellular and molecular levels. Current data suggest that targeting pathways such as BMPs and Wnt signalling is likely to convert bony ankylosis into fibrous ankylosis. Alternatively, promoting MSCs of the radiolucent zone into adipocyte differentiation using FGF21 may be a promising strategy to prohibit the onset of bony ankylosis, and even avoid the occurrence of fibrous ankylosis.

## Abbreviations

ank: Progressive ankylosis; ANKH: Progressive ankylosis gene; BMP: Bone morphogenetic protein; EHPVO: Extrahepatic portal venous obstruction; FGF21: Fibroblast growth factor-21; GNAI3: Guanine nucleotide-binding protein (G protein), α inhibiting activity polypeptide 3; MSCs: Mesenchymal stem cells; PLCB4: Phospholipase C, β4; PPARγ: Peroxisome proliferator-activated receptor-γ; Shox2: Short stature homeobox 2; TGF-β: Transforming growth factor β; TMJ: Temporomandibular joint; Wnt: Wingless-related MMTV integration site.

## Competing interest

The authors declare that they have no competing interests.

## Authors’ contributions

YBY drafted the manuscript and wrote the text. SXL drafted the manuscript and helped with writing the text. JS and JCZ drafted the manuscript and reviewed it critically. YZ revised the final version of the manuscript. All authors read and approved the final manuscript.

## Authors’ information

Yan YB is an Associate Clinical Professor at the Department of Oral and Maxillofacial Surgery of Tianjin Stomatological Hospital. Liang SX is an Associate Clinical Professor at the Department of Operative Dentistry and Endodontics of Tianjin Stomatological Hospital. Shen J is a Professor at the Department of Oral and Maxillofacial Surgery of Tianjin Stomatological Hospital. Zhang JC is a Clinical Professor at the Department of Oral and Maxillofacial Surgery of Tianjin Stomatological Hospital. Zhang Y is a Head Professor at the Department of Oral and Maxillofacial Surgery of Peking University School and Hospital of Stomatology.
